# Post-Brexit no-trade-deal scenario: Short-term consumer benefit at the expense of long-term economic development

**DOI:** 10.1371/journal.pone.0237500

**Published:** 2020-09-03

**Authors:** Leonie Wenz, Anders Levermann, Sven Norman Willner, Christian Otto, Kilian Kuhla

**Affiliations:** 1 Potsdam Institute for Climate Impact Research, Potsdam, Germany; 2 Mercator Research Institute on Global Commons and Climate Change, Berlin, Germany; 3 Department of Agriculture and Resource Economics, University of California, Berkeley, CA, United States of America; 4 Columbia University, New York, NY, United States of America; 5 Institute of Physics, University of Potsdam, Potsdam, Germany; Scuola Superiore Sant'Anna, ITALY

## Abstract

After the United Kingdom has left the European Union it remains unclear whether the two parties can successfully negotiate and sign a trade agreement within the transition period. Ongoing negotiations, practical obstacles and resulting uncertainties make it highly unlikely that economic actors would be fully prepared to a “no-trade-deal” situation. Here we provide an economic shock simulation of the immediate aftermath of such a post-Brexit no-trade-deal scenario by computing the time evolution of more than 1.8 million interactions between more than 6,600 economic actors in the global trade network. We find an abrupt decline in the number of goods produced in the UK and the EU. This sudden output reduction is caused by drops in demand as customers on the respective other side of the Channel incorporate the new trade restriction into their decision-making. As a response, producers reduce prices in order to stimulate demand elsewhere. In the short term consumers benefit from lower prices but production value decreases with potentially severe socio-economic consequences in the longer term.

## Introduction

If the Brexit transition periods ends without the United Kingdom and the European Union having agreed on a trade deal and arranged an orderly transition, trade across the English Channel is likely to undergo a sudden shock due to e.g. procedural complications at the borders [1, 2]. Although some preparation is underway, the ongoing negotiations, practical difficulties and resulting uncertainties make it highly unlikely that the administration and the private sector are prepared for the sudden implementation of required customs procedures. Here, we provide a numerical market shock simulation for a scenario in which the UK leaves the European single market and customs union at the end of the transition without a trade deal (referred to as “no-trade-deal event” hereafter) to shed light on its economic effects. These simulations are by no means a literal prediction of the full socio-economic consequences of Brexit as such a prediction is impossible. They can, however, serve as qualitative insights with quantitative predictive power limited to the order of magnitude of the economic response. We restrict our simulations to the 30 days following a no-trade-deal event.

The likely possibility of a relatively unprepared customs situation in the course of a no-trade-deal event requires numerical simulations with agents that follow a clear economic decision rationale in which they can be ‘surprised’ in the sense that they have informed expectations but do not foresee the future [3]. This necessitates a modeling approach that can dissolve economic dynamics at short timescales of days which is difficult to achieve with mainstream economic models such as gravity models, Computable General Equilibrium or Input-Output models that typically have much coarser temporal resolutions or lack dynamics. The theory of shock models has been developed in the context of extreme events and within the sphere of disaster impact studies; partly with focus on multi-regional spillover or short-term effects [4–14]. Here we build on and apply this theory by combining multi-regional Input-Output (MRIO) data with an agent-based modeling approach. MRIO data are well suited for investigating economic interdependencies [15, 16] but have so far only been used for static analyses of different Brexit scenarios [17–20]. Contrary to these works, we here provide a dynamic shock simulation of the days to weeks following a no-trade-deal event. Our numerical modeling framework depicts the economic response to such an event at day-level thereby accounting for important buffer mechanisms and market adjustments in the short-term such as stockpiling, use of idle capacities, and shifting of demand to unaffected suppliers [21]. We thus complement previous work that focused on longer-term adaptation to a Post-Brexit market (e.g. new trade deals [22]), analyzed the economic effects of a specific trade agreement [23] or identified key industries in the UK-EU trade relationships [20].

We use the numerical loss-propagation model Acclimate that was specifically designed to simulate the short-term global repercussions of unexpected local shocks [24]. It can be used to study the direct and indirect economic effects of a local disruption of infrastructure or productive capacity. Indirect effects thereby denote the spillover of economic losses and gains to initially unaffected sectors and regions because of ripple effects in the supply chain including price and demand changes [25]. The Acclimate model has, for example, been used for computations of future economic losses due to river flood extremes and heat-stress-induced production failure [26, 27]. At its core is the interaction of more than 6,600 heterogeneous economic agents, i.e. regional sectors (referred to as firms hereafter) and consumers, with more than 1.87 million flows between them, to account for the complex effects arising in trade networks [28]. In each time step, these economic agents form explicit expectations on the future. Based on these expectations, each agent then individually decides upon its optimal production level and upon its optimal strategy of distributing demand for input goods among suppliers by maximizing its future expected profit. Importantly, agents have only limited fore- and oversight. Their decisions reflect the information available to them at the time of decision-making and are not the result of an intertemporal optimization procedure. Since we assume a demand-driven economy, agents do not produce more than has been requested. Without external perturbation, the economy is assumed to be in equilibrium, i.e. supply matches demand. This baseline situation, which serves as our counterfactual, is given by the Eora MRIO data [29] that have been used for economic impact analysis in various other studies (e.g. [30, 31]).

Within the Acclimate modeling framework we simulate a no-trade-deal scenario by restricting trade between the UK and the remaining 27 EU countries from one day to the next and for the whole simulation period of 30 days. More precisely, we assume that only a certain percentage of the amount of goods that were previously traded between the UK and the EU can pass through in the days to weeks following a no-trade-deal event, referred to as ‘*border permeability’* hereafter. This reduced border permeability does not mean that all trade is completely cut off nor does it represent the introduction of specific tariffs. Instead, it is supposed to mimic a relatively unprepared customs situation with e.g. administrative obstacles at the borders and harbors that result in delays such that only a certain amount of all previously traded goods transits in the same amount of time.

In total, we distinguish between four scenarios of border permeability and simulate the economic consequences during the first 30 days following the end of the transition period for a scenario in which no trade agreement has been agreed upon by the EU and the UK. We then analyze the time evolution of production and consumption in the UK, the EU and 240 other countries and regions around the globe during this period. Our simulation results highlight the importance of depicting economic response dynamics to market shocks such as the UK leaving the European single market without trade agreement on the same time scale as these events occur. We find that the value of both, production and consumption is reduced at the end of the simulation period and that this decrease is due to demand-side effects that occur immediately after the end of the transition period if no trade deal is put into place. These drops in demand and the resulting decreases in production and prices have received little attention compared to supply-side effects of Brexit (such as shortages of goods) and could aggravate the overall socioeconomic implications.

## Materials and methods

### Economic data

**Fig 1:**
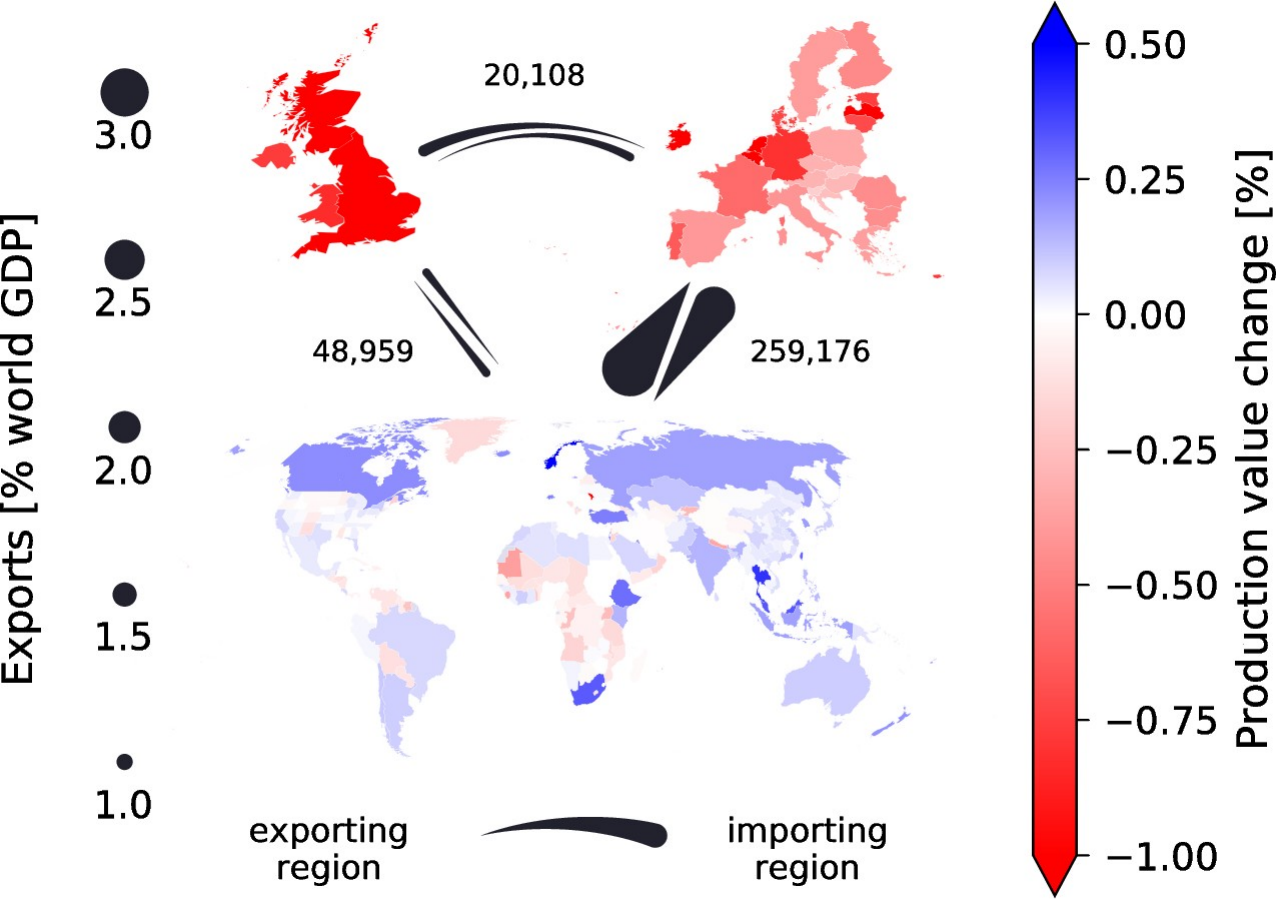
Production value changes after 30 days of a no-trade-deal event and trade relations between the UK, the EU and the rest of the world according to the underlying 2012 multi-regional Input-Output data. Maps show all countries and administrative units covered by our analysis. Thickness of arrows indicates how much is exported from one region to the other. Numbers above arrows state the number of trade connections between these two regions. In our simulations, we assume that trade between the UK and the EU is restricted to a certain percentage of the baseline values throughout the simulation period. Shading of colors is according to production value changes after 30 days of reduced border permeability. Changes shown here refer to a scenario of 70% border permeability. Complementary maps displaying changes in production quantities and prices after 30 days of a no-trade-deal event can be found in the Supplementary Materials (S2 Fig in S1 File).

For the description of the global trade network, we use multi-regional Input-Output data in basic prices for the year 2012 as provided by the Eora World MRIO simplified dataset (v199.82 [32, 33]). These data describe annual monetary flows between 26 sectors and final demand in 188 countries. We interpret them as a measure of quantitative flows. Assuming that economic output is evenly generated throughout the year, we generate mean daily economic flow data (in USD). Using a disaggregation algorithm [34], we obtain state-level resolution for the USA and province-level resolution for China. Thus, the three biggest economies in the world–EU, China and the USA–are represented in a comparably detailed manner. In addition, we disaggregate the UK into England, Scotland, Wales and Northern Ireland which yields a total of 271 regions across the world (Fig 1 and S1 Table in S1 File). Because small flows are more likely to be subjected to large balancing adjustments in the data generating process [32], flows smaller than 1 Million USD (per year) are neglected in the computation. We thus obtain a globe-spanning network of daily economic flows between heterogeneous economic agents, i.e. regional sectors (firms) and final demand (consumers). This trade network serves as counterfactual in our simulations.

### The numerical shock model Acclimate

The simulations are carried out with the numerical agent-based shock model Acclimate [24]. It is an anomaly model evolving around the baseline state of the global trade network as given by the MRIO data. Firms and consumers in this network are modeled as myopic agents with adaptive expectations. Their decision rationales are based on local optimization principles, e.g., firms maximize their expected profit to decide upon their production level and consumers maximize expected consumption to decide upon their purchases.

More specifically, every model time step is divided into three subsequent decisions points or sub-steps [24]. In the first sub-step, firms assess how much they should produce in the current time step to maximize their profits. They take possible constraints into account such as limits of productive capacity or limited input availability. The optimal production quantity is derived by maximizing the difference between revenues and costs. Since we assume a demand-driven economy, revenues are known and reflect how much the firm’s suppliers requested in the last time step and which prices they are willing to pay (reservation prices). Those suppliers that ordered at the highest unit prices are most likely to receive the full amount of goods they requested. In the second sub-step, firms form expectations for the next time step. Based on the demand requests they received in the last time step they estimate which production quantity might be profit-maximizing in the next time step. They then communicate corresponding offer prices, i.e. prices that customers will likely have to offer in order to receive the same amount of goods as before. This helps customers to correct their prices if they deviated hugely from the overall market situation. In the last sub-step, firms assess how many inputs they will presumably need to keep their production going at the optimal level, taking the amount of stockpiled goods into account. They then decide on the cost-minimizing way of distributing this demand among their suppliers under consideration of the respective offer prices that were just communicated. Similarly to firms, consumers decide upon their optimal consumption level, upon the optimal way of distributing their demand and upon the optimal reservation prices.

The model explicitly accounts for the principal factors determining the short-term flexibilities of production systems [21]: Idle capacities that can be activated in times of high demand, input and transport storages to buffer supply shocks, geographically-derived transport times, and price changes. For the simulations in this paper, sectors’ idle capacities are set to 15% of their baseline output, and input storage sizes are set to 15 days of baseline production which means that agents have enough goods stored to uphold their baseline production/consumption quantity levels for 15 days without additional inflow. The model does not consider a restructuring of the global trade network, i.e., agents can only interact along trade relations that are already established. Furthermore, substitution of input goods is not possible. Acclimate is a deterministic model and also in the simulations we adhere to one set of parameters which do not represent probability distributions. A range of likely outcomes can be examined via variation in the forcing. As detailed below, we here consider four different scenarios of border permeability to span a range of likely outcomes of a no-trade-deal event. Moreover, we conduct two additional analyses, each with a slightly different interpretation of a no-trade-deal event.

As detailed in refs. [24, 35], the Acclimate model differs from related modeling approaches such as Computable General Equilibrium (CGE) or Input-Output (IO) models in three important respects. First, the production system in Acclimate is more flexible than what IO models assume but more rigid than the highly flexible production systems in CGE approaches. Second, IO and CGE models are either static in the sense that they compare two different states or, in the case of dynamic CGEs, have coarse time steps of 5 to 10 years. Acclimate, to the contrary, operates on the timescale of days. Consequently, it can represent disequilibrium situations with local demand-supply mismatches that may occur in the immediate aftermath of an economic shock. Importantly, the agents in Acclimate are myopic and cannot foresee these shocks. This is the third major difference to dynamic CGEs with intertemporal optimization which assume perfect foresight of rational agents.

### Modeling of Brexit

We simulate a no-trade-deal event after the transition period by assuming that trade flows between agents in the UK and agents in the 27 remaining EU member states are from one day to the next and for the whole simulation period restricted to a certain percentage of their baseline values. In our main modeling specification, this restriction applies to both, service and commodity sectors but we also provide results for a scenario where solely commodity flows are impacted by the trade restriction (see S2 Table in S1 File for an overview of service and commodity sectors). Furthermore, we conduct an additional analysis where trade flows between the UK and countries with which the EU has trade agreements are restricted as well. For our main modeling specification as well as for each of the two variants, we carry out four simulations with different values of border permeability between 70% and 85% (i.e. 70%, 75%, 80%, and 85%), meaning that only this percentage of the unperturbed trade volume can pass through. We consider different scenarios of trade flow restriction as it is impossible to anticipate the exact difficulties that will arise due to the no-trade-deal situation. Furthermore, we suppose that all economic agents have enough goods stored to uphold their baseline production/consumption quantity levels for 15 days without additional inflow. By embodying enhanced stockpiling of input/consumption goods, we account for some possible preparation measures.

### Production and consumption quantity, prices and values

We look at changes in production and consumption quantities, prices and values over a period of 30 days (compared to the baseline state). The baseline production quantity of a firm is derived from the MRIO data by summing over all its outgoing flows. Production “value” denotes the product of the quantity that is produced and the price for which it is sold. Without loss of generality, all prices are assumed to equal one in the baseline state. In the presence of trade distortions, the value of a firms’ production may change due to changes in the produced quantity or due to changes in prices. Similarly, consumption “value” denotes the product of the quantity that is actually consumed and its price. A negative consumption value change can hence indicate both, that consumption of goods decreases in terms of quantities (because of supply shortages and/or higher prices), or that goods are purchased at lower prices.

The Acclimate model accounts for one representative consumer in each region. Consumed goods are assumed to be perfect complements and consumption changes isoelastically with the consumer price. For each good, price elasticities are set to -0.5.

## Results

For each scenario of reduced border permeability, we assess the short-term economic consequences within the UK, the EU, and the rest of the world by computing the temporal evolution of production and consumption during the first 30 days following a no-trade-deal event. We find that the qualitative results are independent of the specific value of border permeability. The direct economic response in the aftermath of Brexit follows a scenario-independent temporal evolution which strongly supports our confidence in the qualitative insights and in the order of magnitude of the results obtained here. For lower levels of border permeability, daily production seems to “fidget” around a general trend. This is due to the fact that agents have adaptive expectations, i.e. they update their supply and demand decisions at day level under consideration of the gain in information. The magnitude of this fidgeting is more pronounced for scenarios that deviate more strongly from the agents’ baseline and it decreases with the aggregation level of the quantities considered (e.g. when aggregating across sectors of a country and/or across countries of a region). In case of consumption, the consumption elasticities smoothen the consumption response curves.

### Economic effects in the UK and the EU

**Fig 2:**
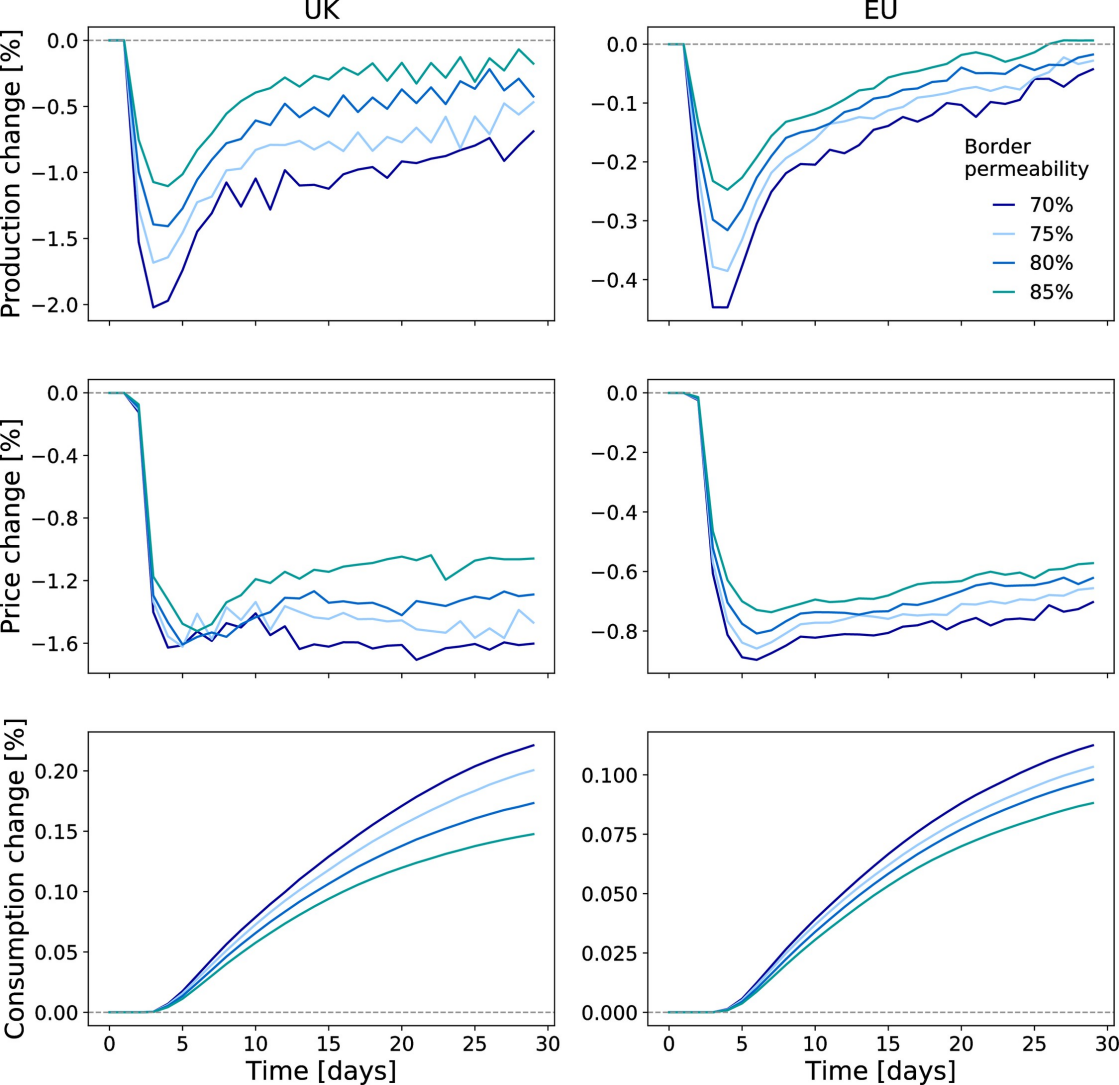
Changes in production, prices and consumption in the UK and the EU during the 30 days following a no-trade-deal event. The number of goods being produced declines in both, the UK and the EU shortly after a no-trade-deal event (first row) because customers on the respective other side of the UK-EU border demand less. Consequently, firms reduce production prices (second row). These lower prices stimulate demand for products slightly. Consumers respond to lower prices; consumption (quantities) rise (third row). In terms of value, i.e. the product of prices and quantities, production and consumption however remain below pre-Brexit levels throughout the simulation period (shown in S1 Fig in S1 File). All changes are depicted as relative deviation from the respective baseline values. Different scenarios of border permeability show similar dynamics. Reduced border permeability is assumed throughout the simulation period.

**Fig 3:**
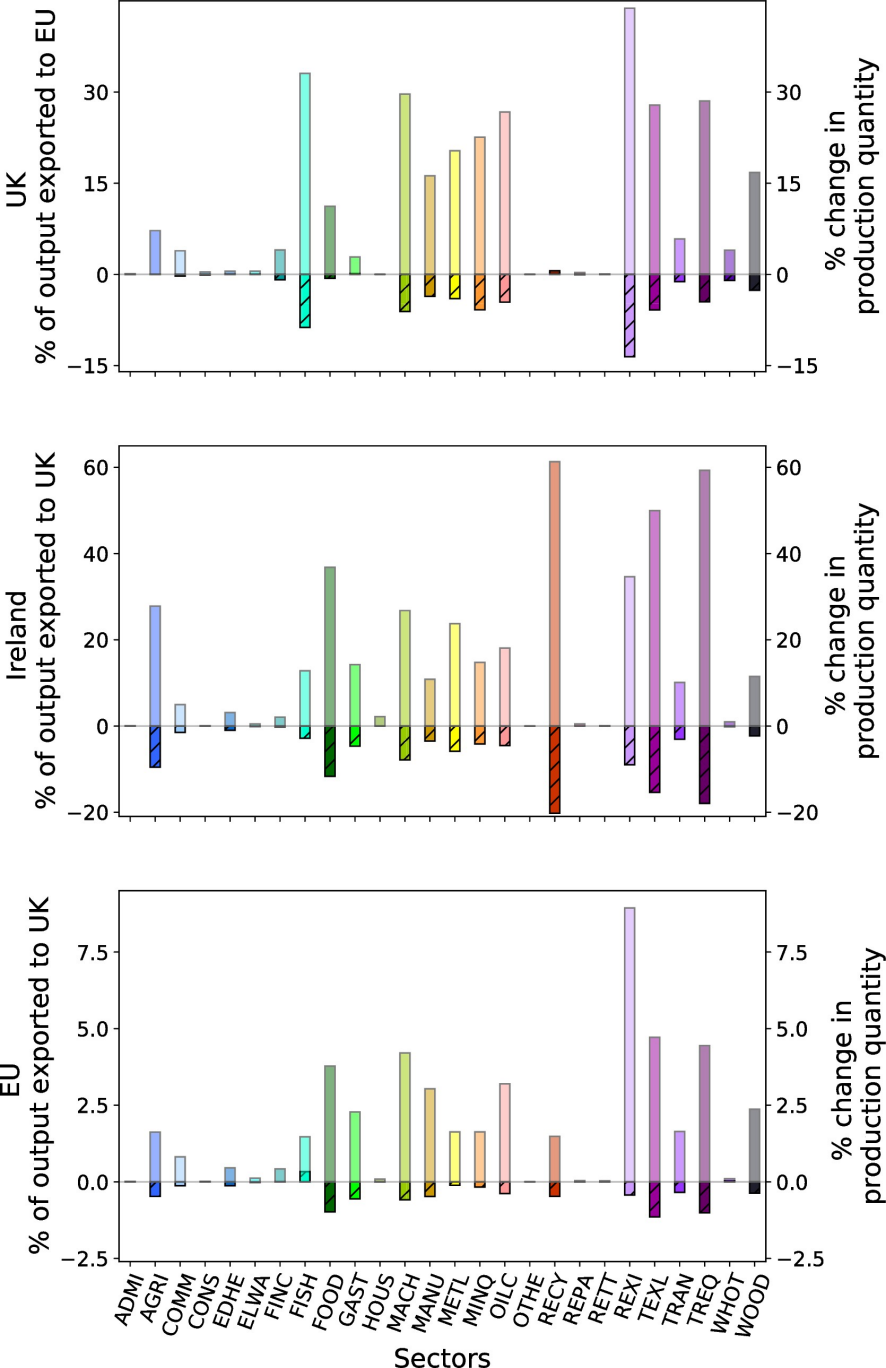
Sectors exporting a large share of their output to customers directly affected by a post-Brexit no-trade-deal event are particularly prone to production losses. Transparently colored bars indicate how much of each sector’s output was exported to the UK/EU in the baseline according to the underlying 2012 multi-regional Input-Output data. Hatched bars show production quantity changes after two days of reduced border permeability. Sectors that heavily rely on sales market on the other side of the UK/EU-border experience in general higher production losses. All sector abbreviations are spelled out in S2 Table in S1 File.

Specifically, we find a drop in the number of goods being produced in the UK and the EU immediately after a no-trade-deal event (Fig 2, first row). These short-term production losses cannot be due to supply shortages because at this point in time firms still have enough input goods in storage to keep their production going. They are, by contrast, *demand-side effects*. Firms on both sides of the English Channel have to reduce their production because they receive less demand from their customers on the respective other side of the Channel. These customers have realized that only a certain amount of the products they requested from their British/EU suppliers can pass through the newly established trade border. They hence decided to demand less from their Brexit-affected suppliers and instead refer to their inventories and other suppliers (compare S1 Fig in S1 File, first row). We find particularly high production losses in sectors that used to export a large share of their output to trading partners that are now on the other side of the UK-EU border (Fig 3).

**Fig 4:**
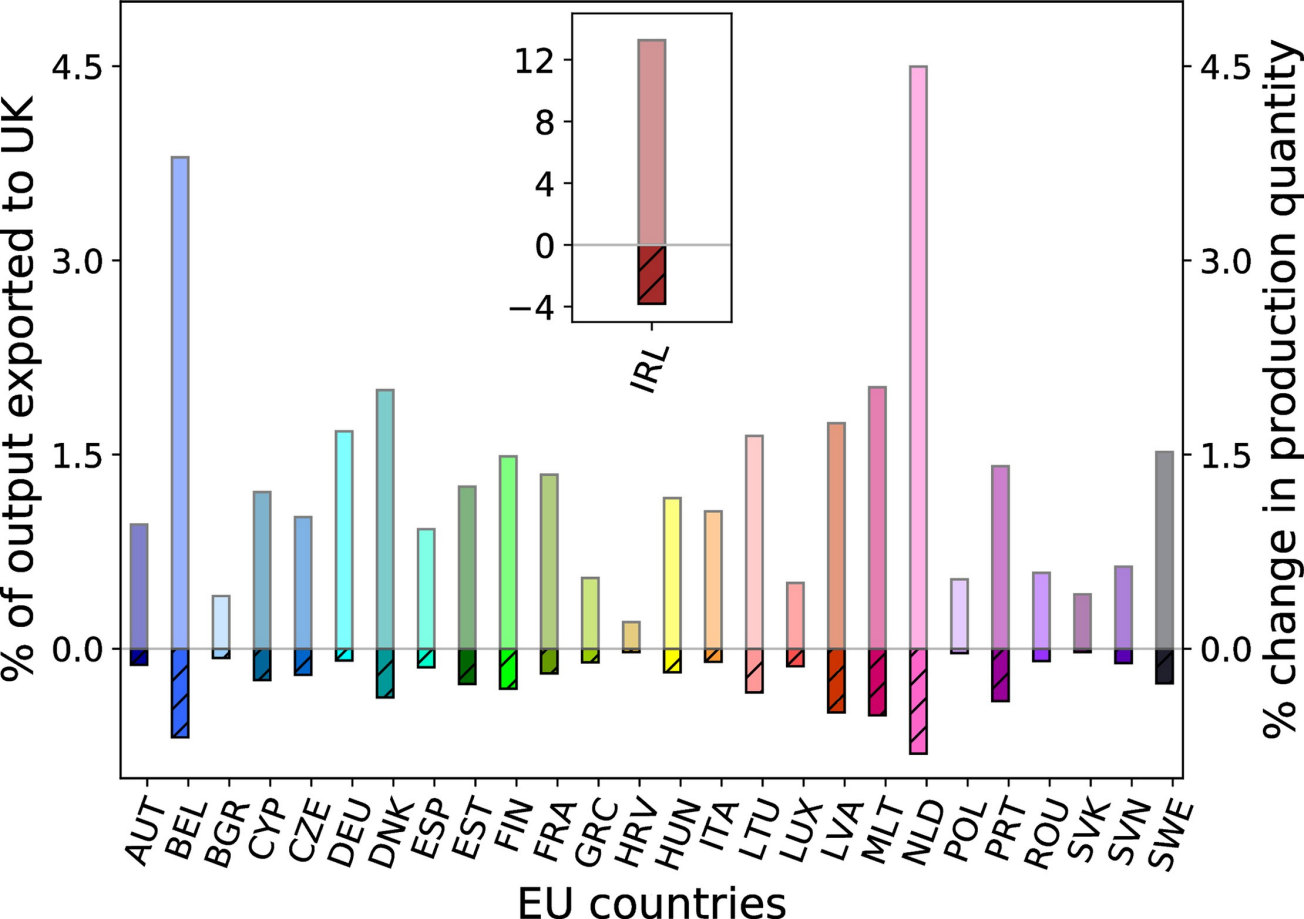
EU countries exporting a large share of their output to the UK experience highest production losses. Transparently colored bars indicate how much of each country’s output was exported to the UK/EU in the baseline according to the underlying 2012 multi-regional Input-Output data. Hatched bars show production quantity changes after two days of reduced border permeability. Countries that export larger shares of their output to the UK experience in general higher production losses. Inlay: Close-up for Ireland.

Since, in relative terms, the EU is of higher importance for British exports than the other way around (Fig 1), we find larger production losses for UK sectors than for EU sectors. Ireland on the other hand heavily relies on the UK as sales market for its products and is even more affected than the UK (Fig 4, inlay and S1 Fig in S1 File). Also within the rest of the EU production losses are highest in those countries that previously exported larger shares of their production to the UK such as Belgium or the Netherlands (Fig 4). In response to the decline in demand, firms decide to sell their products at lower prices with the aim of stimulating demand for their products elsewhere (Fig 2, second row, and S1 Fig in S1 File). Consumers in the UK and the EU react to these lower prices by consuming more (Fig 2, third row). However, this increase in domestic consumption cannot offset the decline in demand from customer firms on the respective other side of the EU-UK border. Even though the amount of goods being produced starts recovering, prices do not rise accordingly and the value of production and consumption, i.e. the product of quantities and prices, remains below baseline levels throughout the simulation period. (S1 Fig in S1 File).

### Economic effects on the rest of the world

In our main simulations, we take a conservative approach in assuming that trade flows between the UK and countries with which the EU has trade agreements are not restricted. Countries outside the EU however can be indirectly affected–through up-stream and down-stream ripple effects in the supply chain as well as through the associated price signals. We find that several countries benefit from a no-trade-deal event in the sense that the value of their production increases (Fig 1 and S1 Fig in S1 File). This applies in particular to the non-EU European countries Iceland, Norway and Switzerland as well as to several Commonwealth countries, e.g. Canada, Australia, India and South Africa. These countries are all in a good position to replace Brexit-affected suppliers due to strong existing trade relations. In most of these countries more goods are produced at slightly lower prices (S2 Fig in S1 File). After firms in the UK and the EU have reduced their prices, prices also fall in most of the other countries around the world (S1 Fig in S1 File) with the notable exception, again, of Norway and Switzerland (S2 Fig in S1 File). Conversely, in some countries, for instance in sub-Saharan Africa, the decline in prices is not offset by a respective increase in production quantities or production quantities even decline resulting in reduced production values (compare Fig 1 and S2 Fig in S1 File).

**Fig 5:**
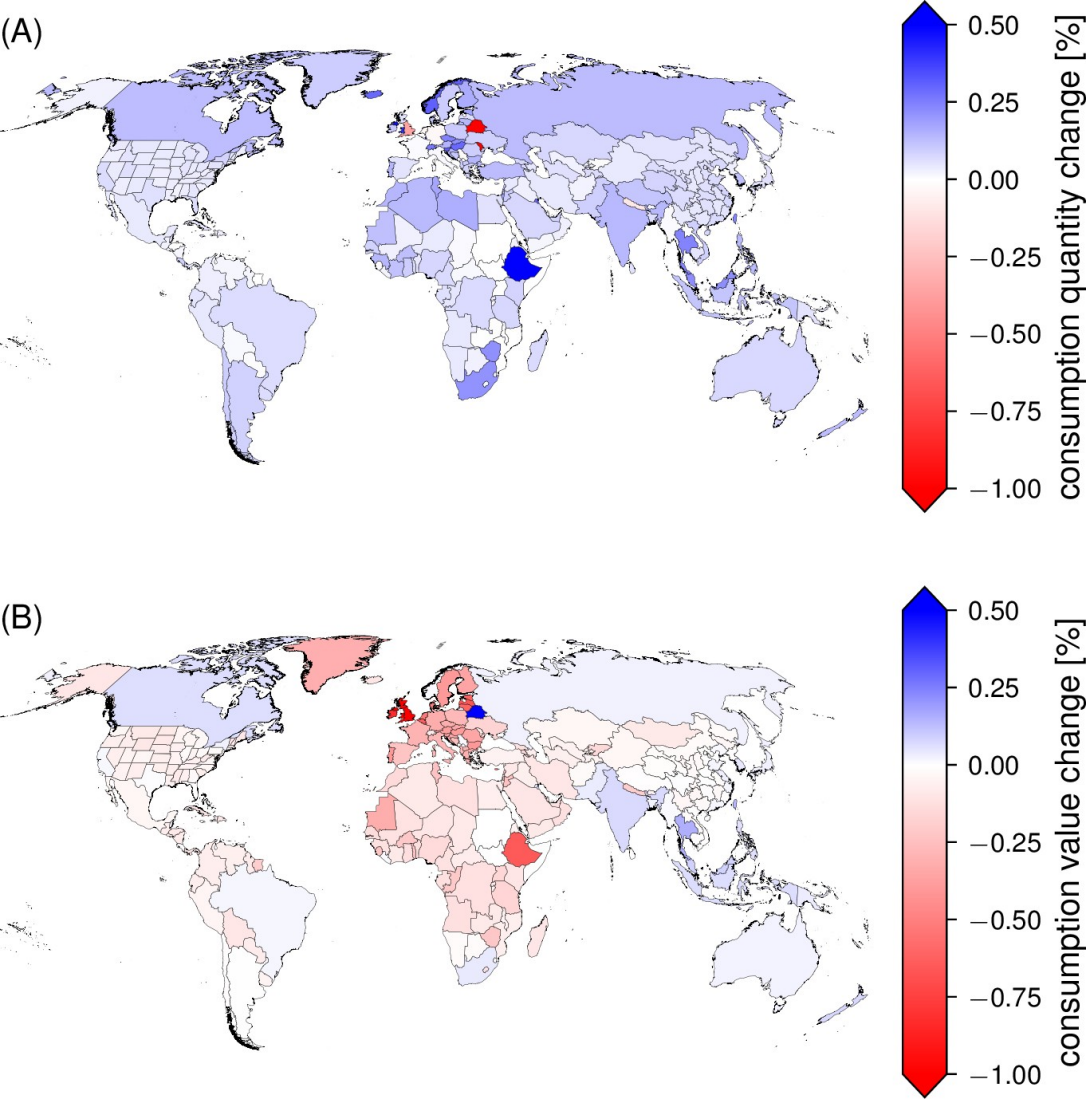
World map of consumption quantity and value changes after 30 days of a no-trade-deal event. Shading of colors is according to consumption quantity (panel A) and consumption value (panel B) changes after 30 days of reduced border permeability. Underlying scenario assumes 70% border permeability. Changes in consumers’ prices are shown in S3 Fig in S1 File.

In terms of consumption changes, the amount of goods being consumed increases slightly in most of the countries (Fig 5, panel A). As firms around the world offer their goods at lower prices, people in many countries consume more in quantitative terms (S1 and S3 Figs in S1 File). However, the value of consumption remains below the baseline level in the majority of countries throughout the simulation period (Fig 5, panel B). Among those countries that benefit from a no-trade-deal event in terms of gains in consumption values are again several Commonwealth countries such as Canada or India.

### Results for variants of a no-trade-deal post-Brexit event

In additional analyses, we consider two variants of our main modeling specification. First, we assume that only commodity sectors are affected by the no-trade-deal event as service sectors typically do not have to cross a geographical border. Second, we do not only restrict trade flows between the UK and EU member countries but also between the UK and countries with which the EU currently has trade agreements (see S1 Table in S1 File for a list). We do not include countries where trade agreements are only partly in place according to the European Commission (as to November 2019) such as Canada or Ukraine. In this model set-up, we again restrict trade flows of both, commodity and service sectors. The results for these two additional variants of a no-trade-deal event are qualitatively similar to those of our main modeling specification (S4 and S5 Figs in S1 File). If service sectors are not affected by the trade restriction, we see the most notable difference in simulation results for Ireland where production quantity is reduced less during the first days of trade restriction (S4 Fig in S1 File). Consecutively, production and consumption prices and values decline to a lesser extent as well whereas consumption (quantity) does not increase as much as in the main modeling specification. In the rest of the EU, the decline in consumption prices is also more moderate than in the main modeling specification.

In case the trade restriction also applies to all flows between the UK and countries with which the EU currently holds trade agreements, the effects on the UK are generally stronger (S5 Fig in S1 File). During the first days of trade restriction, production quantity, price and value decrease more than under the main modeling specification. As a consequence, consumption prices and value are more strongly reduced as well whereas consumption quantity increases more. Ireland, to the contrary, is a little less affected in this modeling specification. In the rest of the world, we observe less positive effects than in the main modeling specification. Here, production quantities do not go up as much in response to the trade restriction suggesting that producers in the rest of the world step in less for UK and EU firms. Consequently, prices fall. In the main modeling specification, production value in the rest of the world increases for all scenarios of border permeability. Here, the picture is less clear. On a more disaggregated level, we see that in particular countries with which the EU has trade agreements such as Norway, Switzerland, Iceland, South Africa, or Japan are now negatively affected in terms of production value changes (S6 Fig in S1 File). These countries were among the main beneficiaries in the main modeling specification.

## Discussion

The major economic incision that a no-trade-deal event as conceptualized by our simulations presents to the UK and Ireland is likely to impact society profoundly. The short-term adverse effects on production we observe here can have a long-lasting impact on economic growth and development. Even though consumers benefit at first from declining prices, the unsustainable economic situation created by the UK leaving the European single market without a trade agreement is likely to affect them negatively in the long-run, e.g. due to labor market effects. The latter might also deepen intra-regional inequality. In general, an economic recession of this magnitude impedes social, ecological and infrastructural projects and innovation. Our simulations are restricted to a period of 30 days following a no-trade-deal event– a period, in which stockpiling is likely to buffer major supply shortages. These supply shortages might, however, hit the already struggling economy at a later point in time thus aggravating the overall situation.

Our simulations can naturally only depict certain aspects of a post-Brexit no-trade-deal scenario and can hence not provide a quantitatively accurate prediction of its real-world consequences. For instance, in our modeling framework we assume that trade between Ireland and the EU is not perturbed. In reality, a large amount of the goods traded between these two partners passes through the UK. As a consequence, our simulations might underestimate the effect of a no-trade-deal event on Ireland and some European economies. Furthermore, our simulations are based on MRIO data for the year 2012. Since the structure of the global trade network changes over time [16, 26] with e.g. new trade agreements being negotiated [36], this represents a potential limitation. For example, the EU has established new or reinforced existing trade relations with several countries since 2012 via regional or bilateral trade agreements. Even though we consider these trade agreements in the additional model variant where trade between the UK and EU trade agreement partners is also affected, these agreements are likely not reflected in the structure of the global trade and supply network as given by the 2012 data. As a consequence, the countries in question might be affected to a stronger extent (positively or negatively) by a no-trade-deal event between the UK and the EU than our simulations predict. Yet, MRIO data are published with some time delay and the 2012 Eora data have the advantage of having already been used and tested in published work [37–39].

Finally, given the high level of complexity of modern-day economies with e.g. nested production processes [28], there are many “unknown unknowns” related to a no-trade-deal event that our modeling framework may or may not capture. Using a network approach that explicitly models the sectoral and regional interlinkages in the global trade and supply network allows us to study cascade effects that can be induced by an economic disintegration of the British economy from the EU single market. In particular, our modeling exercise illuminates an aspect of a no-trade-deal post-Brexit scenario that has received little attention so far and that could worsen its expected overall economic and societal implications. That is the shrinking of potential sales market on both sides of the Channel. We find that these short-term demand-side effects decrease the value of production and consumption and are hence likely to have a substantial negative impact on the British, Irish and, to a lesser degree, European economies.

## Supporting information

S1 File (PDF)

S2 File (ZIP)
