# Supplementary material for: Post-Brexit no-trade-deal scenario: Short-term consumer benefit at the expense of long-term economic development
Source: PLoS One. 2020 Sep 3;15(9):e0237500. doi: 10.1371/journal.pone.0237500 (PMC7470266; doi:10.1371/journal.pone.0237500)
Supplement: S1 File — (PDF) [file pone.0237500.s001.pdf]

Supplementary Information

Brexit no-trade-deal scenario: short-term consumer benefit at the expense of long-term economic development

This document provides supplementary tables and figures to Wenz, Levermann, Willner, Otto & Kuhla (2020).

Supplementary Tables

S1 Table: **Countries and administrative units included in simulations.** Superscripts indicate that region belongs to the United Kingdom (UK), the European Union (EU), the United States of America (USA), China (CHN) or the Commonwealth of Nations (COMM). They also denote whether a country has a trade agreement with the EU thereby distinguishing between different types of agreement: Stabilisation and Association Agreement (SAA), Association Agreement (AA), Customs Union (CU), Partnership and Cooperation Agreement (PCA), Economic Partnership Agreement (EPA), Economic Area Agreement (EAA), Global Agreement (GA), Free Trade Agreement (FTA), Agreement (TA), Interim Association Agreement (IAA), Cooperation Agreement (COA) and Cooperation and Partnership Agreement (CPA). Source: European Commission (https://ec.europa.eu/trade/policy/countries-and-regions/negotiations-and-agreements/).

Afghanistan
Albania[SAA]
Algeria[AA]
Andorra[CU]
Angola
Antigua and Barbuda[COMM]
Argentina
Armenia[PCA]
Aruba
Australia[COMM]
Austria[EU]
Azerbaijan
Bahamas[COMM]
Bahrain
Bangladesh[COMM]
Barbados[COMM]
Belarus
Belgium[EU]
Belize[COMM]
Benin
Bermuda
Bhutan
Bolivia
Bosnia and Herzegovina[SAA]
Botswana[EPA, COMM]
Brazil
British Virgin Islands
Brunei[COMM]
Bulgaria[EU]
Burkina Faso
Burundi
Cambodia
Cameroon[COMM]
Canada[COMM]
Cape Verde
Cayman Islands
Central African Republic
Chad
Chile[AA]
Anhui[CHN]
France[EU]
French Polynesia
Gabon
Gambia[COMM]
Georgia[AA]
Germany[EU]
Ghana[COMM]
Greece[EU]
Greenland
Guatemala
Guinea
Guyana[COMM]
Haiti
Honduras
Hong Kong
Hungary[EU]
Iceland[EAA]
India[COMM]
Indonesia
Iran
Iraq
Ireland[EU]
Israel[AA]
Italy[EU]
Jamaica[COMM]
Japan[EPA]
Jordan[AA]
Kazakhstan
Kenya[COMM]
Kuwait
Kyrgyzstan
Laos
Latvia[EU]
Lebanon[AA]
Lesotho[EPA, COMM]
Liberia
Libya
Liechtenstein[EAA]
Lithuania[EU]
Luxembourg[EU]
Slovakia[EU]
Slovenia[EU]
Somalia
South Africa[EPA, COMM]
South Sudan
Spain[EU]
Sri Lanka[CPA, COMM]
Sudan
Suriname
Swaziland[COMM]
Sweden[EU]
Switzerland[TA]
Syria[COA]
Taiwan
Tajikistan
Thailand
Macedonia[SAA]
Togo
Trinidad and Tobago[COMM]
Tunisia[AA]
Turkey[CU]
Turkmenistan
Uganda[COMM]
Ukraine
United Arab Emirates
England[UK]
Northern Ireland[UK]
Scotland[UK]
Wales[UK]
Tanzania[COMM]
Alabama[USA]
Alaska[USA]
Arizona[USA]
Arkansas[USA]
California[USA]
Colorado[USA]
Connecticut[USA]
Delaware[USA]
District of Columbia[USA]
Florida[USA]

Beijing
Chongqing[CHN]
Fujian[CHN]
Gansu[CHN]
Guangdong[CHN]
Guangxi[CHN]
Guizhou[CHN]
Hainan[CHN]
Hebei[CHN]
Heilongjiang[CHN]
Henan[CHN]
Hubei[CHN]
Hunan[CHN]
Jiangsu
Jiangxi
Jilin
Liaoning
Nei Mongol
Ningxia Hui
Qinghai
Shaanxi
Shandong
Shanghai
Shanxi
Sichuan
Tianjin
Xinjiang Uygur
Xizang
Yunnan
Zhejiang
Colombia
Republic of Congo
Costa Rica
Croatia[EU]
Cuba
Cyprus[EU]
Czech Republic[EU]
Côte d'Ivoire
North Korea
Democratic Republic of the Congo
Denmark[EU]
Djibouti
Dominican Republic
Ecuador
Egypt[AA]
El Salvador
Eritrea
Estonia[EU]
Ethiopia
Fiji[COMM]
Finland[EU]
Macao
Madagascar
Malawi[COMM]
Malaysia[COMM]
Maldives
Mali
Malta[EU, COMM]
Mauritania
Mauritius[COMM]
Mexico[GA]
Monaco
Mongolia
Montenegro[SAA]
Morocco[AA]
Mozambique[EPA, COMM]
Myanmar
Namibia[EPA, COMM]
Nepal
Netherlands[EU]
Netherlands Antilles
New Caledonia
New Zealand[COMM]
Nicaragua
Niger
Nigeria[COMM]
Norway[EAA]
Palestina[IAA]
Oman
Pakistan[COMM]
Panama
Papua New Guinea[COMM]
Paraguay
Peru
Philippines
Poland[EU]
Portugal[EU]
Qatar
South Korea[FTA]
Moldova[AA]
Romania[EU]
Russia
Rwanda[COMM]
Samoa[COMM]
San Marino[CU]
São Tomé and Príncipe
Saudi Arabia
Senegal
Serbia[SAA]
Seychelles[COMM]
Sierra Leone[COMM]
Singapore[COMM]
Georgia[USA]
Hawaii[USA]
Idaho[USA]
Illinois[USA]
Indiana[USA]
Iowa[USA]
Kansas[USA]
Kentucky[USA]
Louisiana[USA]
Maine[USA]
Maryland[USA]
Massachusetts[USA]
Michigan[USA]
Minnesota[USA]
Mississippi[USA]
Missouri[USA]
Montana[USA]
Nebraska[USA]
Nevada[USA]
New Hampshire[USA]
New Jersey[USA]
New Mexico[USA]
New York[USA]
North Carolina[USA]
North Dakota[USA]
Ohio[USA]
Oklahoma[USA]
Oregon[USA]
Pennsylvania[USA]
Rhode Island[USA]
South Carolina[USA]
South Dakota[USA]
Tennessee[USA]
Texas[USA]
Utah[USA]
Vermont[USA]
Virginia[USA]
Washington[USA]
West Virginia[USA]
Wisconsin[USA]
Wyoming[USA]
Uruguay
Uzbekistan
Vanuatu[COMM]
Venezuela
Vietnam
Yemen
Zambia[COMM]
Zimbabwe

S2 Table: **Sectors included in the simulation.** In the main modeling specification, we assume that commodity and service sectors are both affected by a no-trade-deal event between the EU and the UK. In a further model variant, service sectors are assumed to be unaffected by reduced permeability of the UK-EU border. Abbreviations for sectors as used in Fig. 3 are given in brackets.

Commodity sectors	Service sectors
Agriculture (AGRI)	Public Administration (ADMI)
Construction (CONS)	Post & Telecommunications (COMM)
Financial Intermediation & Business Activities (FINC)	Education, Health & Other Services (EDHE)
Fishing (FISH)	Electricity, Gas & Water (ELWA)
Food & Beverages (FOOD)	Hotels & Restaurants (GAST)
Electrical & Machinery (MACH)	Private Households (HOUS)
Other Manufacturing (MANU)	Others (OTHE)
Metal Products (METL)	Recycling (RECY)
Mining & Quarrying (MINQ)	
Petroleum, Chemical & Non-Metallic Mineral Products (OILC)	
Maintenance & Repair (REPA)	
Retail Trade (RETT)	
Re-export & Re-import (REXI)	
Textiles & Wearing Apparel (TEXL)	
Transport (TRAN)	
Transport Equipment (TREQ)	
Wholesale Trade (WHOT)	
Wood & Paper (WOOD)	

Supplementary Figures

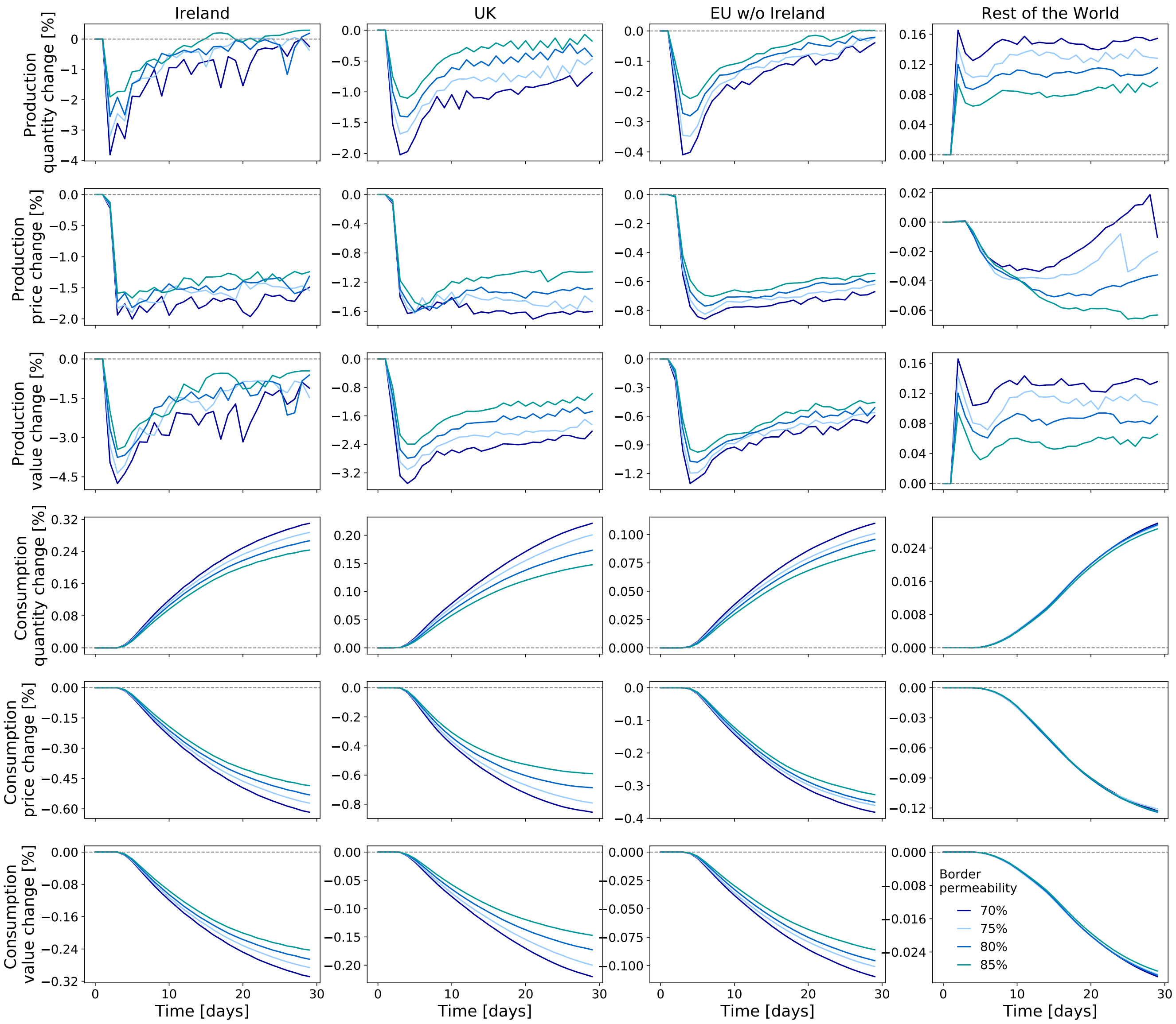

S1 Fig: **Changes in production and consumption quantities, prices and values in Ireland, the UK, the remaining EU and the rest of the world during the 30 days following a no-trade-deal event (main modeling specification).** Different scenarios of border permeability show similar dynamics. Reduced border permeability is assumed throughout the simulation period. In response to drops in production quantities in the UK and the EU, production prices and consumer prices fall. Even though lower prices stimulate demand for products slightly, production and consumption values remain below baseline levels throughout the simulation period. In the rest of the world, production slightly increases as customers request more from their suppliers that are not affected by the trade restriction. All changes are depicted as relative deviation from the respective baseline levels.

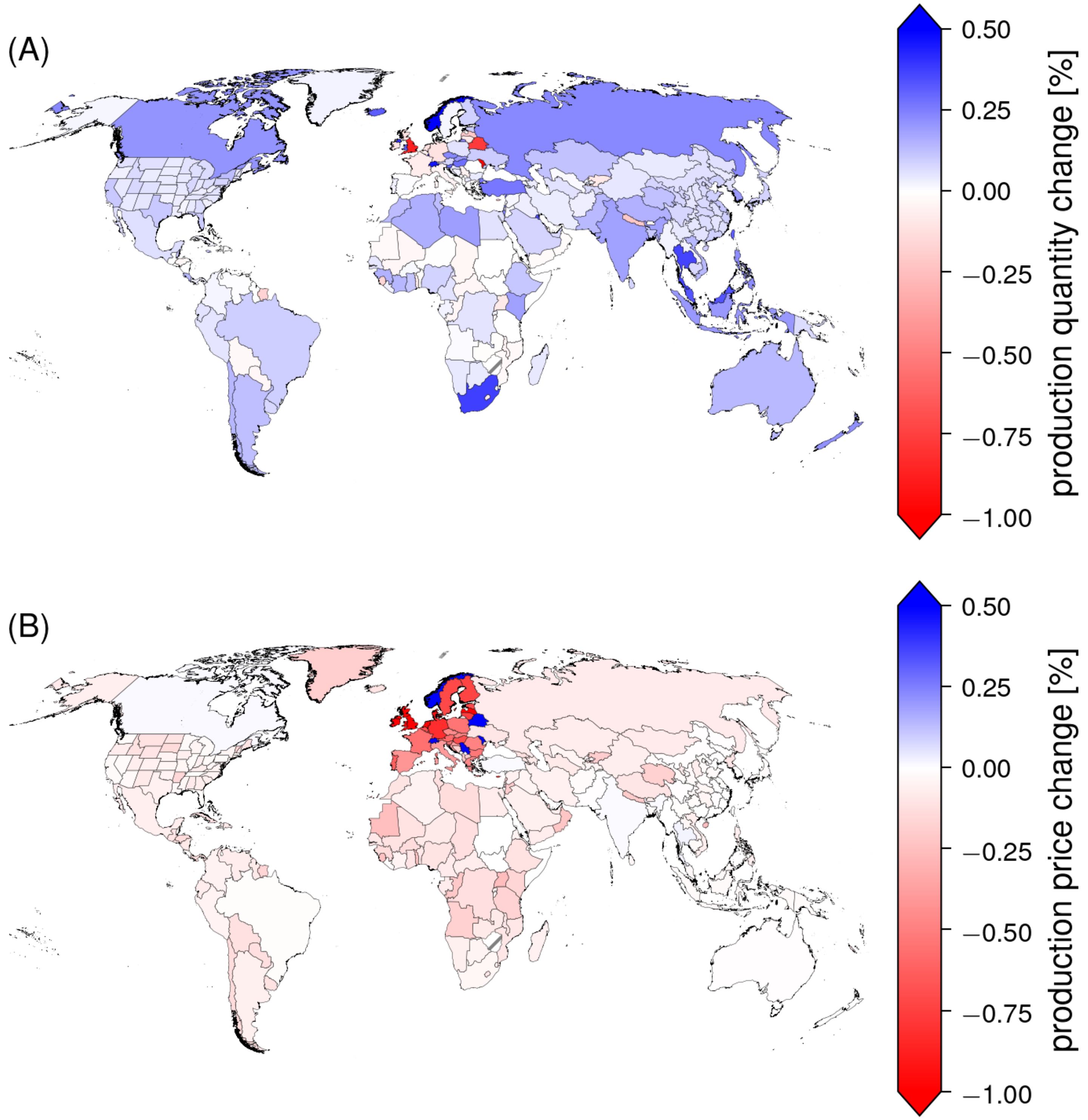

S2 Fig: **World map of production quantity and price changes after 30 days of a no-trade-deal event (main modeling specification).** Shading of colors is according to production quantity and production price changes after 30 days of reduced border permeability. Underlying scenario assumes 70% border permeability. Changes in production value are shown in Fig. 1 in main manuscript.

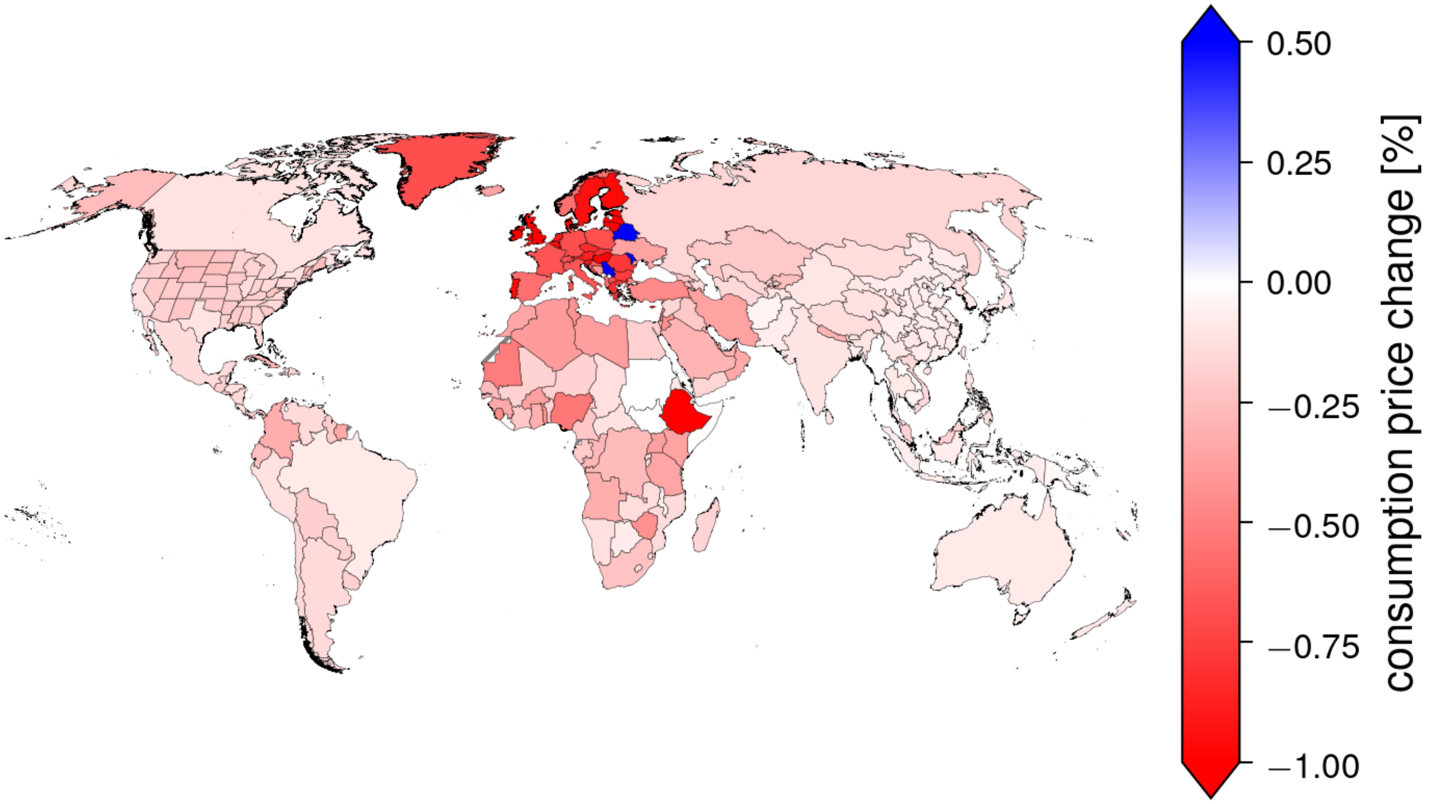

S3 Fig: **World map of consumption price changes after 30 days of a no-trade-deal event (main modeling specification).** Shading of colors is according to consumption price changes after 30 days of reduced border permeability. Underlying scenario assumes 70% border permeability. Changes in consumption quantity and consumption value are shown in Fig. 5 in main manuscript.

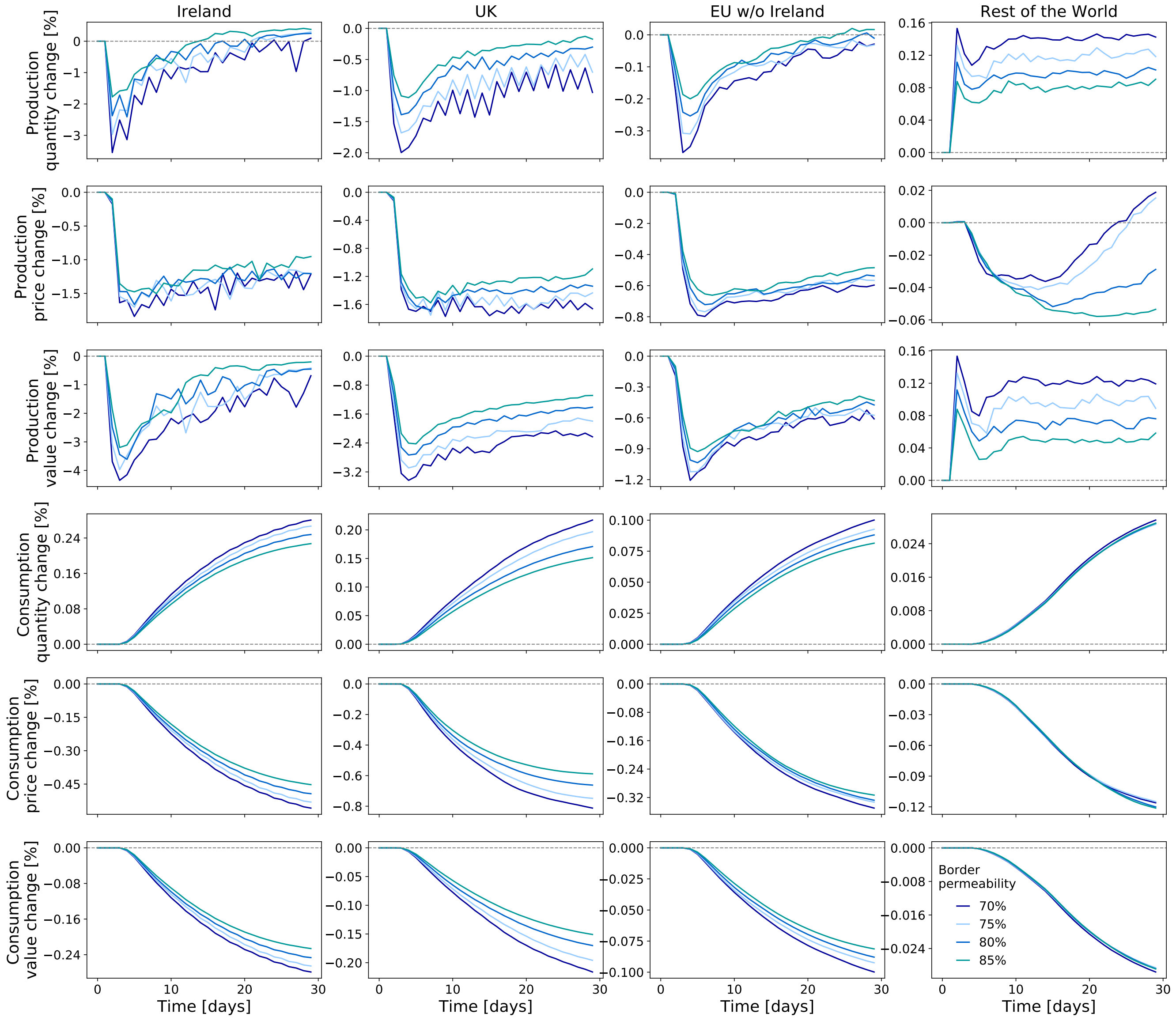

S4 Fig: **Changes in production and consumption quantities, prices and values in Ireland, the UK, the remaining EU and the rest of the world during the 30 days following a no-trade-deal event (alternative modeling specification #1).** Simulation results for alternative modeling specification where only commodity sectors but not service sectors are affected by the trade restriction between the UK and the EU. Overall dynamics are very similar to those of the main modeling specification. Most notably, the negative effect on Ireland is stronger than in the main modeling specification. All changes are depicted as relative deviation from the respective baseline levels.

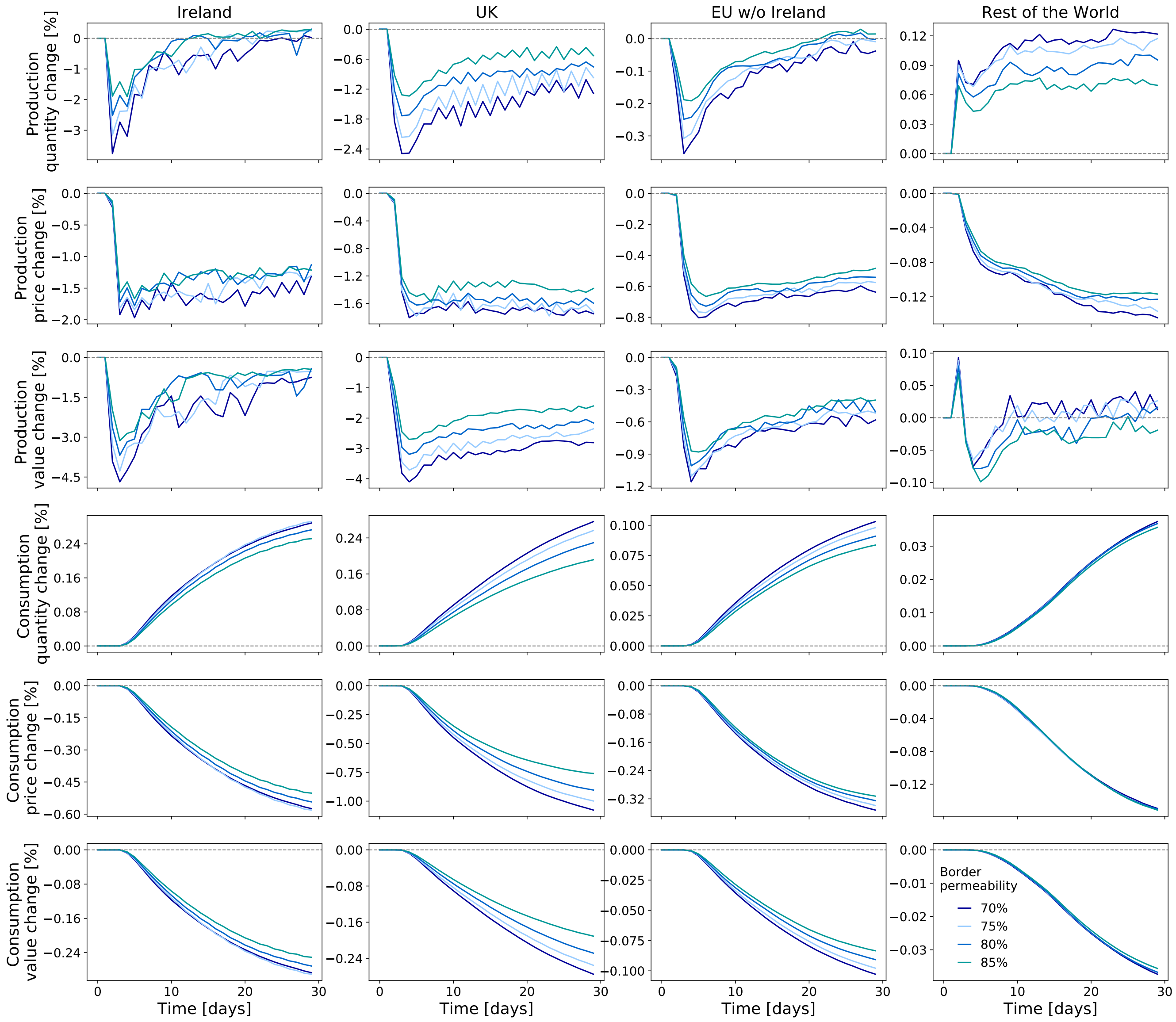

S5 Fig: **Changes in production and consumption quantities, prices and values in Ireland, the UK, the remaining EU and the rest of the world during the 30 days following a no-trade-deal event (alternative modeling specification #2).** Simulation results for alternative modeling specification where trade between the UK and countries with which the EU has trade agreements is also restricted. Overall dynamics are very similar to those of the main modeling specification. Most notably, the negative impact on the UK is stronger whereas the positive effects in the rest of the world are less pronounced. All changes are depicted as relative deviation from the respective baseline levels.

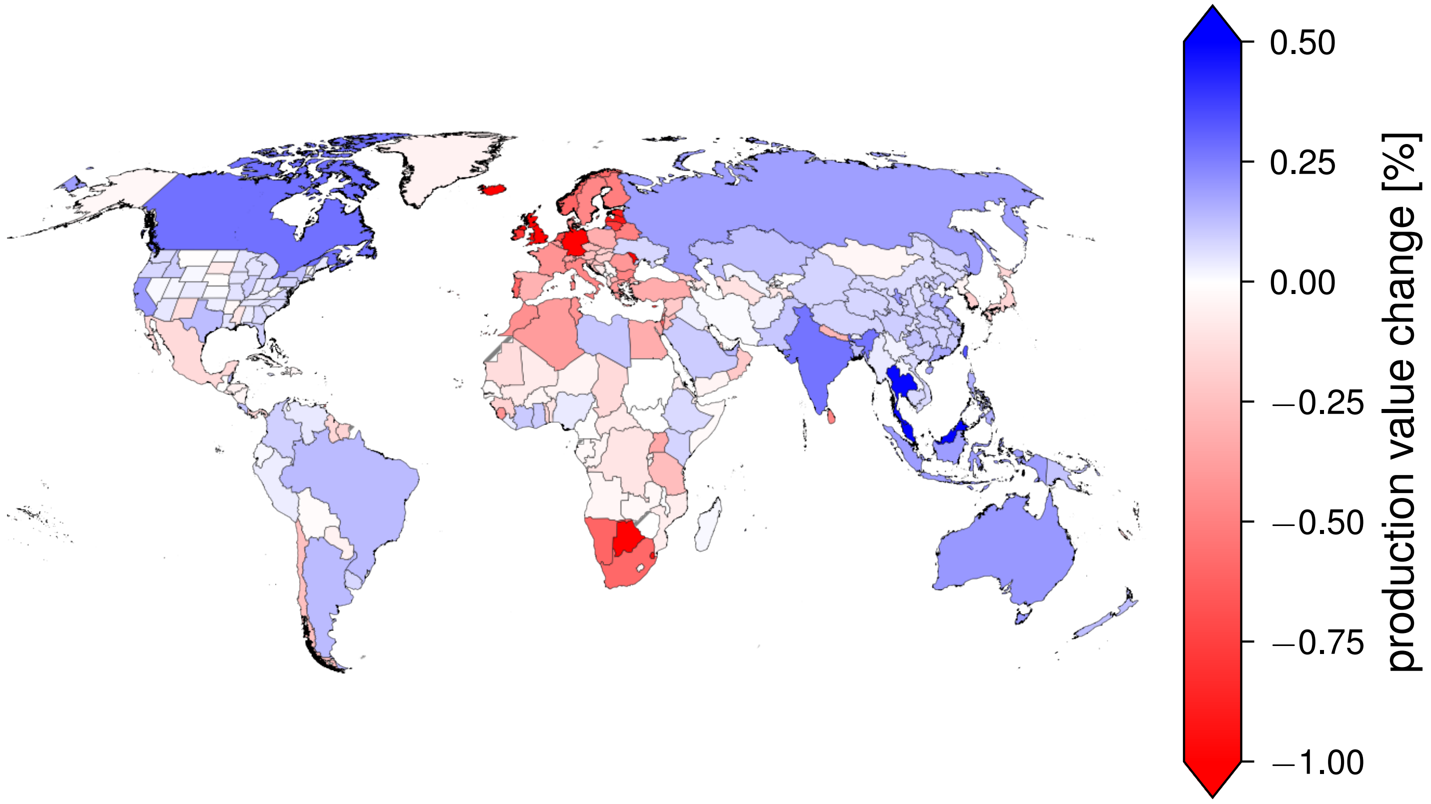

S6 Fig: **World map of production value changes after 30 days of a no-trade-deal event (alternative modeling specification #2).** Production value changes in many of the EU trading partner countries are reversed (from positive in the main modeling specification to negative here). Shading of colors is according to consumption price changes after 30 days of reduced border permeability. Underlying scenario assumes 70% border permeability.
